# Clinical features and death risk factors in COVID-19 patients with cancer: a retrospective study

**DOI:** 10.1186/s12879-021-06495-9

**Published:** 2021-08-05

**Authors:** Yi Zhou, Qiao Yang, Jun Ye, Xiaocheng Wu, Xianhua Hou, Yimei Feng, Bangyu Luo, Jixi Li, Guangrong Yang, Lingchen Li, Xiu Yang, Bin Wang, Songtao Zhao, Li Li, Qi Li, Zhi Xu, Hao Wu, Jianguo Sun

**Affiliations:** 1 Cancer Institute, Xinqiao Hospital, Army Medical University, 83 Xinqiao Zhengjie Road, Shapingba, Chongqing, 400037 People’s Republic of China; 2 Department of Ultrasound, The 941st Hospital of the PLA Joint Logistic Support Force, Xining, People’s Republic of China; 3 Department of Gastroenterology, Southwest Hospital, Army Medical University, Chongqing, People’s Republic of China; 4 Department of Emergency, Xinqiao Hospital, Army Medical University, Chongqing, People’s Republic of China; 5 Department of Neurology, Southwest Hospital, Army Medical University, Chongqing, People’s Republic of China; 6 Department of Hematology, Xinqiao Hospital, Army Medical University, Chongqing, People’s Republic of China; 7 Department of Respiratory and Intensive Care, Xinqiao Hospital, Army Medical University, 83 Xinqiao Zhengjie Road, Shapingba, Chongqing, 400037 People’s Republic of China; 8 Institute of Infectious Diseases, Southwest Hospital, Army Medical University, Chongqing, People’s Republic of China; 9 Department of Respiratory Medicine, Army Medical Center, Chongqing, People’s Republic of China; 10 Xinqiao Hospital, Army Medical University, 83 Xinqiao Zhengjie Road, Shapingba, Chongqing, 400037 People’s Republic of China

**Keywords:** COVID-19, Cancer, Nomogram, Neutrophil-to-lymphocyte ratio, C-reactive protein

## Abstract

**Background:**

Coronavirus disease 2019 (COVID-19) has spread around the world. This retrospective study aims to analyze the clinical features of COVID-19 patients with cancer and identify death outcome related risk factors.

**Methods:**

From February 10th to April 15th, 2020, 103 COVID-19 patients with cancer were enrolled. Difference analyses were performed between severe and non-severe patients. A propensity score matching (PSM) analysis was performed, including 103 COVID-19 patients with cancer and 206 matched non-cancer COVID-19 patients. Next, we identified death related risk factors and developed a nomogram for predicting the probability.

**Results:**

In 103 COVID-19 patients with cancer, the main cancer categories were breast cancer, lung cancer and bladder cancer. Compared to non-severe patients, severe patients had a higher median age, and a higher proportion of smokers, diabetes, heart disease and dyspnea. In addition, most of the laboratory results between two groups were significantly different. PSM analysis found that the proportion of dyspnea was much higher in COVID-19 patients with cancer. The severity incidence in two groups were similar, while a much higher mortality was found in COVID-19 patients with cancer compared to that in COVID-19 patients without cancer (11.7% vs. 4.4%, P = 0.028). Furthermore, we found that neutrophil-to-lymphocyte ratio (NLR) and C-reactive protein (CRP) were related to death outcome. And a nomogram based on the factors was developed.

**Conclusion:**

In COVID-19 patients with cancer, the clinical features and laboratory results between severe group and non-severe group were significantly different. NLR and CRP were the risk factors that could predict death outcome.

**Supplementary Information:**

The online version contains supplementary material available at 10.1186/s12879-021-06495-9.

## Background

Coronavirus disease 2019 (COVID-19) has spread around the world. Patients with COVID-19 presented with fever, cough, dyspnea, vomiting and diarrhea, and the critical patients might have acute respiratory distress syndrome and multiorgan failure. Analysis of the blood test results of the patients showed that the neutrophil counts, lymphocyte counts and biochemical indexes were abnormal and the pro-inflammatory cytokines and infection-related biomarkers were highly expressed [1, 2]. People with diabetes [3], hypertension [4], and heart disease [5] were thought to be susceptible to COVID-19 and had poor clinical outcome. Cancer patients were considered to have lower immunity than normal people. Therefore, they were susceptible to infected with bacteria and viruses [6, 7]. Among patients with advanced cancer, 20% had lymphopenia [8]. Therefore, immunosuppression status of cancer patients put them at an increased risk of COVID-19.

In the early stage (before February 2020) of COVID-19 outbreak in Wuhan, China, medical staff and materials were inadequate, leading to a high mortality rate. By February 2020, with the arrival of a large number of medical supplies and more than 40,000 medical workers in Wuhan, the patients with COVID-19 got timely treatment, and the mortality reduced gradually [9]. Some studies reported that cancer patients were more susceptible to COVID-19 and more likely to deteriorate into a severe form [9–12]. However, most of the cases they reported were in the early stage of the outbreak, and there was a lack of knowledge and response measures of the disease. In this study, we collected cases admitted to hospital in the late stage of the outbreak in Wuhan, China, reflecting the real status of cancer patients with COVID-19 whom were admitted under adequate treatment. We aimed to determine the risk factors of disease severity and death, and sought to develop a nomogram to predict the risk.

## Methods

### Patients selection

This study was approved by the Ethics Committee of Taikang Hospital (TKTJLL-007). The Ethics Committee of the Taikang Hospital waived the need for informed consent of each patient. From February  10th to April 15th, 2020, a total of 2980 patients with confirmed COVID-19 infection by RT-PCR were enrolled from Taikang hospital and other temporary hospitals of Wuhan in this retrospective study, including 103 cancer patients. All patients were divided into severe or non-severe group according to the Diagnosis and Treatment Protocol for COVID-19 (Trial Version 7) published by the National Health Commission of China. The severe patients were defined as following: the respiratory rate is more than 30 breaths per minute, the oxygen saturation is lower than 93% in rest state, oxygenation index is no greater than 300 mmHg, respectively. Propensity score matching (MatchIt package, R, version 3.5.2) was used to match each COVID-19 patient with cancer with two COVID-19 patients without cancer for further analysis. The following predetermined factors were considered: age, gender and comorbidities, such as diabetes, hypertension and heart disease. The flow chart of the study design was shown in Additional file 1: Figure S1.

### Data collection and difference analyses

Clinical features, and laboratory results of all patients with COVID-19 were obtained from the hospitals through the way of desk review of patient medical records. All data were extracted manually. Clinical features included demographics, comorbidities and symptoms. The laboratory examinations included routine blood tests, inflammation-related biomarkers, renal and liver function, biochemical tests, and coagulation function. Difference analyses between 103 COVID-19 patients with cancer and 206 matched COVID-19 patients without cancer were performed. In addition, we performed difference analyses between severe and non-severe groups, as well as between survivors and non-survivors in COVID-19 patients with cancer.

### Development and validation of a nomogram

First of all, factors were excluded if missing values of the factor reached more than 20%. Then, missing values were imputed using multivariate imputation if factors had missing values less than 20%. Generalized linear model was used to select significant risk factors (P < 0.05) that affect death outcome. A nomogram was developed with significant risk factors. The area under the curve (AUC) of the receiver operating characteristic (ROC) curve, calibration plot, decision curve and clinical impact curve were used to validate the nomogram. R packages used in this step including MICE, pROC, rmda, regplot, rms and PredictABEL (version 3.5.2).

### Statistical analysis

Continuous variables were expressed by median and interquartile range (IQR), and categorical variables were represented as frequencies. All statistical analyses were performed using SPSS (version 17.0). The Mann–Whitney U test was used to compare continuous data and Pearson’s Χ^2^ test used to compare contingency data. A two-sided P value < 0.05 was considered statistically significant.

## Results

### Cancer distribution in 103 COVID-19 patients

A total of 103 (3.5%) cancer cases in 2980 patients with COVID-19 were enrolled in this study. The cancer categories of the 103 patients included breast cancer (22.3%), lung cancer (16.5%), bladder cancer (8.7%), esophageal cancer (7.8%), gastric cancer (6.8%), thyroid cancer (5.8%), rectal cancer (4.9%), colon cancer (3.9%), larynx cancer (3.9%), cervical cancer (3.9%), prostate cancer (3.9%), liver cancer (2.9%), nasopharyngeal cancer (2.9%), endometrial cancer (2.9%), renal cancer (1%), ovarian cancer (1%) and testicular cancer (1%) (Table1).

**Table 1:** The categories of the 103 COVID-19 patients with cancer

Tumor types	Number of patients (%)
Breast cancer	23 (22.3%)
Lung cancer	17 (16.5%)
Bladder cancer	9 (8.7%)
Esophagus cancer	8 (7.8%)
Gastric cancer	7 (6.8%)
Thyroid cancer	6 (5.8%)
Rectal cancer	5 (4.9%)
Cervical cancer	4 (3.9%)
Larynx cancer	4 (3.9%)
Colon cancer	4 (3.9%)
Prostate cancer	4 (3.9%)
Nasopharyngeal cancer	3 (2.9%)
Liver cancer	3 (2.9%)
Endometrial cancer	3 (2.9%)
Testicular cancer	1 (1%)
Ovarian cancer	1 (1%)
Renal cancer	1 (1%)

### Comparison between severe and non-severe patients

The patients consisted of 47 females (45.6%) and 56 males (54.4%), and 36 (35%) in severe group and 67 (65%) in non-severe group. The median age of cancer patients was 66 (ranging from 24 to 90), and the age in the severe group was older than that in non-severe group. The smoking people accounted for a higher proportion in the severe group than in the non-severe group (Table 2).

**Table 2:** Clinical features and laboratory findings differences between severe and non-severe patients

Clinical features	All (n = 103)	Non-severe (n = 67)	Severe (n = 36)	P-value
Age	66.0 (24.0–90.0)	65.0 (43.0–90.0)	70.0 (24.0–87.0)	0.004
Gender				0.097
Female	47 (45.6%)	35 (52.2%)	12 (33.3%)	
Male	56 (54.4%)	32 (47.8%)	24 (66.7%)	
Smoking	20 (19.4%)	8 (11.9%)	12 (33.3%)	0.017
Comorbidities				
Diabetes	14 (13.6%)	5 (7.5%)	9 (25%)	0.018
Hypertension	37 (35.9%)	22 (32.8%)	15 (41.7%)	0.396
Heart disease	11 (10.7%)	4 (6.0%)	7 (19.4%)	0.047
Symptoms				
Fever	73 (70.9%)	45 (67.2%)	28 (77.8%)	0.363
Cough	70 (68.0%)	49 (73.1%)	21 (58.3%)	0.183
Dyspnea	24 (23.3%)	9 (13.4%)	15 (41.7%)	0.003
Vomiting	5 (4.9%)	2 (3.0%)	3 (8.3%)	0.340
Diarrhea	7 (6.8%)	3 (4.5%)	4 (11.1%)	0.235
Laboratory findings				
WBC (× 10^9^/L)	5.29 (1.90–25.10); n = 100	4.77 (1.90–25.10); n = 65	7.16 (2.00–18.80); n = 35	< 0.001
Neutrophil (× 10^9^/L)	3.49 (0.67–22.40); n = 100	3.00 (0.67–22.40); n = 65	5.27 (1.03–17.36); n = 35	< 0.001
Lymphocyte (× 10^9^/L)	1.06 (0.22–2.89); n = 100	1.23 (0.33–2.62); n = 65	0.80 (0.22–2.89); n = 35	0.003
NLR	2.93 (0.64–32.23); n = 100	2.29 (0.64–18.39); n = 65	6.93 (1.20–32.23); n = 35	< 0.001
Monocytes (× 10^9^/L)	0.43 (0.11–9.90); n = 72	0.41 (0.11–9.90); n = 52	0.50 (0.14–1.04); n = 20	0.297
Eosnophils (× 10^9^/L)	0.07 (0.00–0.90); n = 72	0.08 (0.00–0.51); n = 52	0.03 (0.00–0.90); n = 20	0.007
Basophils (× 10^9^/L)	0.02 (0.00–0.11); n = 72	0.02 (0.00–0.11); n = 52	0.02 (0.00–0.05); n = 20	0.09
RBC (× 10^12^/L)	3.95 (2.03–5.77); n = 73	4.01 (2.03–5.14); n = 52	3.53 (2.63–5.77); n = 21	0.116
HB (g/L)	116.00 (68.00–154.00); n = 73	119.00 (68.00–154.00); n = 52	102.00 (79.00–154.00); n = 21	0.065
PLT (× 10^9^/L)	203.00 (53.00–431.00); n = 72	205.00 (67.00–431.00); n = 52	190.00 (53.00–312.00); n = 20	0.606
CRP (mg/L)	2.52 (0.14–280.32); n = 90	1.39 (0.14–137.52); n = 58	42.59 (0.05–280.32); n = 32	< 0.001
ALT (U/L)	19.17 (4.50–131.20); n = 97	19.05 (4.50–131.20); n = 64	20.70 (6.00–100.30); n = 33	0.87
AST (U/L)	21.70 (10.00–147.20); n = 84	20.45 (10.00–147.20); n = 52	26.70 (12.00–92.60); n = 32	0.014
Total protein (g/L)	64.99 (53.70–87.58); n = 71	65.50 (55.45–87.58); n = 51	60.89 (53.70–78.97); n = 20	0.04
Albumin (g/L)	37.29 (23.60–48.00); n = 71	38.14 (23.60–48.00); n = 51	32.61 (25.30–43.50); n = 20	0.002
Globulin (g/L)	28.00 (17.31–46.24); n = 71	27.30 (17.31–40.49); n = 51	28.20 (24.00–46.24); n = 20	0.818
A/G	1.30 (0.39–2.20); n = 71	1.35 (0.39–2.20); n = 51	1.22 (0.71–1.46); n = 20	0.017
Total bilirubin (umol/L)	10.32 (4.20–44.58); n = 70	10.23 (4.20–33.46); n = 51	11.70 (6.00–44.58); n = 19	0.152
Direct Bilirubin (umol/L)	2.71 (0.00–23.90); n = 70	2.50 (0.00–14.20); n = 51	3.86 (0.00–23.90); n = 19	0.053
Indirect bilirubin (umol/L)	7.39 (2.99–26.42); n = 70	7.32 (3.00–26.42); n = 51	7.90 (2.99–22.68); n = 19	0.468
BUN (umol/L)	4.67 (2.68–29.61); n = 68	4.62 (2.68–29.61); n = 49	5.47 (2.93–18.77); n = 19	0.114
Creatinine (umol/L)	57.72 (13.25–345.35); n = 70	58.17 (31.60–345.35); n = 50	55.15 (13.25–145.39); n = 20	0.559
Uric acid (umol/L)	268.01 (100.00–275.95)); n = 69	304.73 (151.56–575.95); n = 50	163.00 (100.00–547.00); n = 19	< 0.001
ALP (U/L)	71.10 (42.59–493.30); n = 70	67.80 (42.59–493.30); n = 51	74.60 (55.16–238.00); n = 19	0.212
γ-GT (U/L)	26.47 (8.15–263.60); n = 70	26.30 (8.15–263.60); n = 51	33.62 (11.02–239.60); n = 19	0.262
CK (U/L)	48.40 (10.90–195.90); n = 55	52.67 (16.89–116.73); n = 37	43.14 (10.90–195.90); n = 18	0.244
CKMB (U/L)	7.80 (0.01–54.60); n = 68	7.61 (0.01–54.60); n = 50	7.85 (2.28–49.70); n = 18	0.416
LDH (U/L)	179.90 (2.17–761.70); n = 69	169.13 (2.17–761.70); n = 50	209.66 (116.20–702.30); n = 19	0.001
ɑ-HBDH (U/L)	133.59 (87.75–701.40); n = 66	126.96 (87.75–701.40); n = 48	169.50 (99.79–604.40); n = 18	0.005
D-dimer (mg/L)	0.43 (0.02–27.94); n = 45	0.37 (0.07–4.97); n = 30	1.14 (0.02–27.94); n = 15	0.001
NT-proBNP (U/L)	30.77 (0.01–515.18); n = 22	11.33 (0.01–112.76); n = 11	60.90 (23.94–515.81); n = 11	0.001
Procalcitonin (ng/ml)	0.05 (0.02–0.90); n = 56	0.04 (0.02–0.45); n = 36	0.18 (0.02–0.90); n = 20	< 0.001
IL-6 (pg/ml)	3.64 (1.50–3392.00); n = 50	2.84 (1.50–268.30); n = 33	29.99 (1.50–3392.00); n = 17	0.001

*WBC* white blood cell, *NLR* leukocyte to lymphocyte ratio, *RBC* red blood cell, *HB* hemoglobin, *PLT* platelet, *CRP* C-reactive protein, *ALT* alanine transaminase, *AST* aspartate transaminase, *A/G* albumin to globulin ratio, *BUN* blood urea nitrogen, *ALP* alkaline phosphatase, *γ-GT* gamma-glutamyl transpeptidase, *CK* creatine kinase, *CKMB* MB isoenzyme of creatine kinase, *LDH* lactate dehydrogenase, *α-HBDH* alpha-hydroxybutyric dehydrogenase, *NT-proBNP* N-terminal pro brain natriuretic peptide, *IL-6* interleukin-6

There were 42 (40.8%) patients who had comorbidities, such as hypertension (35.9%), diabetes (13.6%), and heart disease (10.7%), 14 of whom had two or more comorbidities. Among the 36 severe patients, 18 (50%) cases had comorbidities, while in the 67 non-severe patients 24 (35.8%) had comorbidities. A higher proportion of patients with diabetes or heart disease were observed in the severe group than in the non-severe group (Table 2).

The common clinical symptoms of cancer patients were fever (70.9%), cough (68%), dyspnea (23.3%), diarrhea (6.8%) and vomiting (4.9%). The percentage of patients with cough, vomiting or diarrhea showed no significant difference between the two groups except for a higher percentage of patients had dyspnea in the severe group than in the non-severe group (41.7% vs. 13.4%, P = 0.003) (Table 2).

The median level of white blood cell (WBC) count was 5.29 × 10^9^ /L in all cancer patients, 4.77 × 10^9^ /L in non-severe patients and 7.16 × 10^9^ /L in severe patients. Similar results were found for neutrophil count. The median level of lymphocyte count was significantly lower in the severe group than in the non-severe group (0.80 × 10^9^ /L vs. 1.23 × 10^9^ /L, P = 0.003). In addition, a higher neutrophil-to-lymphocyte ratio (NLR) in severe patients was found (6.93 vs. 2.29, P < 0.001). The level of procalcitonin, C-reactive protein (CRP) and interleukin-6 (IL-6) was higher in severe patients than in non-severe patients (Table 2). When comparing hepatic function, renal function and biochemical indexes between severe and non-severe patients with cancer, we found that lactate dehydrogenase (LDH), alpha-hydroxybutyric dehydrogenase (αHBDH), D-dimer, N-terminal pro brain natriuretic peptide (NTproBNP), aspartate transaminase (AST) and Uric acid were higher while total protein, albumin and albumin/globulin were lower in the severe group (Table 2).

### Propensity score matching analysis

We performed a 1:2 (COVID-19 with cancer: COVID-19 without cancer) matched case–control analysis. Age, gender and comorbidities were used for matching. Then, differences in demographics, comorbidities, symptoms, severity, clinical outcome and laboratory findings were compared (Table 3). We found that COVID-19 patients with cancer had a higher proportion of dyspnea than COVID-19 patients without cancer (23.3% vs. 10.2%, P = 0.003). No other significant differences were found. When comparing laboratory results between two groups, we found that the median lymphocyte count, monocytes count, eosinophils count and alanine transaminase (ALT) were lower in COVID-19 patients with cancer than in COVID-19 patients without cancer (Table 3). In contrast, the median NLR was higher in COVID-19 patients with cancer compared to counterpart. No significant differences were found in other laboratory results. The proportion of severe patients was similar between two groups (35% vs. 31%, P = 0.520). The proportion of deaths was much higher in COVID-19 patients with cancer than in COVID-19 patients without cancer (11.7% vs. 4.4%, P = 0.028) (Table 3).

**Table 3:** Comparison between COVID-19 patients with cancer and without cancer

Clinical features	All patients (n = 309)	Non-cancer patients (n = 206)	Cancer patients (n = 103)	P-value
Age	66.0 (20.0–95.0)	66.00 (20.00–95.00)	66.0 (24.0–90.0)	0.948
Gender				0.810
Female	138 (44.7%)	91 (44.1%)	47 (45.6%)	
Male	171 (55.3%)	115 (55.8%)	56 (54.4%)	
Comorbidities				
Diabetes	37 (11.9)	23 (11.1%)	14 (13.6%)	0.579
Hypertension	96 (31.0%)	59 (28.6%)	37 (35.9%)	0.195
Heart disease	41 (13.2)	30 (14.5%)	11 (10.7%)	0.379
Symptoms				
Fever	234 (74.7%)	161 (78.1)	73 (70.9%)	0.163
Cough	207(66.9%)	137 (66.5%)	70 (68.0%)	0.898
Dyspnea	45 (14.6%)	21 (10.2%)	24 (23.3%)	0.003
Vomiting	10 (3.2%)	5 (2.4%)	5 (4.9%)	0.310
Diarrhoea	17 (5.5)	10 (4.8%)	7 (6.8%)	0.597
Severity				0.520
Non-severe	209 (67.6%)	142 (68.9%)	67 (65%)	
Severe	100 (32.4%)	64 (31%)	36 (35%)	
Clinical outcome				0.028
Survivor	288 (93.2%)	197 (95.6%)	91 (88.3%)	
Non-survivor	21 (6.8%)	9 (4.4%)	12 (11.7%)	
Laboratory findings				
WBC (× 109**/**L)	5.50 (0.80–28.20); n = 306	5.60 (0.80–28.2); n = 206	5.29 (1.90–25.10); n = 100	0.381
Neutrophil (× 109**/**L)	3.25 (0.44–26.07); n = 306	3.23 (0.44–26.07); n = 206	3.49 (0.67–22.40); n = 100	0.164
Lymphocyte (× 109**/**L)	1.41 (0.60–3.50); n = 306	1.54 (0.60–3.50); n = 206	1.06 (0.22–2.89); n = 100	< 0.001
NLR	2.28 (0.20–62.12); n = 306	2.11 (0.20–62.12); n = 206	2.93 (0.64–32.23); n = 100	< 0.001
Monocytes (× 109/L)	0.48 (0.03–1.16); n = 278	0.50 (0.03–1.16); n = 206	0.43 (0.11–1.11); n = 72	0.040
Eosnophils (× 109/L)	0.11 (0.00–0.90); n = 278	0.12 (0.00–0.72); n = 206	0.07 (0.00–0.90); n = 72	< 0.001
Basophils (× 109/L)	0.03 (0.00–0.11); n = 278	0.03 (0.00–0.08); n = 206	0.02 (0.00–0.11); n = 72	0.076
RBC (× 1012/L)	3.93 (1.17–5.77); n = 279	3.93 (1.17–5.60); n = 206	3.95 (2.03–5.77); n = 73	0.406
HB (g/L)	120.00 (53.00–166.00); n = 279	120.50 (53.00–166.00); n = 206	116.00 (68.00–154.00); n = 73	0.074
PLT (× 109**/**L)	209.50 (4.00–443.00); n = 278	213.00 (4.00–443.00); n = 206	203.00 (53.00–431.00); n = 72	0.120
CRP (mg/L)	2.27 (0 .01–280.32); n = 251	2.16 (0.01–268.60); n = 161	2.52 (0.14–280.32); n = 90	0.147
ALT (U/L)	21.67 (4.50–239.70); n = 301	23.35 (5.30–239.70); n = 204	19.17 (4.50–131.20); n = 97	0.047
AST (U/L)	21.55 (9.60–502.40); n = 260	21.51 (9.60–502.40); n = 176	21.70 (10.00–147.20); n = 84	0.374
Total protein (g/L)	65.5 (32.70–87.58); n = 276	65.81 (32.70–81.6); n = 205	64.99 (53.70–87.58); n = 71	0.528
Albumin (g/L)	37.30 (18.3–48.00); n = 276	37.36 (18.30–45.55); n = 205	37.29 (23.60–48.00); n = 71	0.723
Globulin (g/L)	27.73 (14.40–46.24); n = 276	27.70 (14.40–45.91); n = 205	28.00 (17.31–46.24); n = 71	0.417
A/G	1.33 (0.39–2.65); n = 276	1.33 (0.70–2.65); n = 205	1.30 (0.39–2.20); n = 71	0.338
Total bilirubin (umol/L)	10.00 (4.20–415.70); n = 274	9.76 (4.20–415.70); n = 204	10.32 (4.20–44.58); n = 70	0.514
Direct Bilirubin (umol/L)	2.50 (0.00–326.70); n = 274	2.50 (0.00–326.70); n = 204	2.71 (0.00–23.90); n = 70	0.412
Indirect bilirubin (umol/L)	7.18 (2.69–89.01); n = 274	7.17 (2.69–89.01); n = 204	7.39 (2.99–26.42); n = 70	0.883
BUN (umol/L)	4.88 (2.23–36.08); n = 272	4.93 (2.23–36.08); n = 204	4.67 (2.68–29.61); n = 68	0.471
Creatinine (umol/L)	60.00 (13.25–345.35); n = 273	60.87 (26.77–243.40); n = 203	57.72 (13.25–345.35); n = 70	0.509
Uric acid (umol/L)	281.63 (69.00–600.56); n = 273	286.02 (69.00–600.56); n = 204	268.01 (100.00–575.95); n = 69	0.274
ALP (U/L)	71.10 (31.90–493.30); n = 272	71.12 (31.90–366.10); n = 202	71.10 (42.59–493.30); n = 70	0.461
γ-GT (U/L)	26.50 (7.64–263.6); n = 273	26.50 (7.64–242.2); n = 203	26.47 (8.15–263.60); n = 70	0.813
CK (U/L)	44.31 (10.90–1210.00); n = 229	43.20 (11.00–1210.00); n = 174	48.40 (10.90–195.90); n = 55	0.084
LDH (U/L)	175.35 (2.17–1489.89); n = 270	174.10 (99.90–489.89); n = 201	179.90 (2.17–761.70); n = 69	0.686
ɑ-HBDH (U/L)	130.30 (79.66–1028.00); n = 267	129.80 (79.66–1028.00); n = 201	133.59 (87.75–701.40); n = 66	0.511
D-dimer (mg/L)	0.44 (0.01−27.94); n = 172	0.44 (0.01−6.91); n = 127	0.43 (0.02–27.94); n = 45	0.498
NT-proBNP (U/L)	43.94 (0.01–35,000.00); n = 102	51.61 (0.01–35,000.00); n = 80	30.77 (0.01–515.18); n = 22	0.503
Procalcitonin (ng/ml)	0.045 (0.01–87.04); n = 200	0.042 (0.01–87.04); n = 144	0.05 (0.02–0.90); n = 56	0.231
IL-6 (pg/ml)	3.20 (1.50–5000.00); n = 189	3.0 (1.50–5000.00); n = 139	3.64 (1.50–3392.00); n = 50	0.099

*WBC* white blood cell, *NLR* leukocyte to lymphocyte ratio, *RBC* red blood cell, *HB* hemoglobin, *PLT* platelet, *CRP* C-reactive protein, *ALT* alanine transaminase, *AST* aspartate transaminase, *A/G* albumin to globulin ratio, *BUN* blood urea nitrogen, *ALP* alkaline phosphatase, *γ-GT* gamma-glutamyl transpeptidase, *CK* creatine kinase, *CKMB* MB isoenzyme of creatine kinase, *LDH* lactate dehydrogenase, *α-HBDH* alpha-hydroxybutyric dehydrogenase, *NT-proBNP* N-terminal pro brain natriuretic peptide, *IL-6* interleukin-6

### Nomogram for predicting death outcome

As of April 15th, 2020, 12 out of 103 COVID-19 patients with cancer died in this study. To identify risk factors for death outcome and develop a nomogram, we excluded factors if the missing values were more than 20%. Hence, age, gender, smoking, comorbidities, symptoms, WBC count, Neutrophil count, Lymphocyte count, NLR, CRP, ALT and AST were included for further analysis. Comparison between survivors and non-survivors were performed. As shown in Table 4, when comparing clinical features, a higher proportion of dyspnea was found in non-survivors. No significant differences were found in other clinical features between two groups. When comparing laboratory results, we found that WBC count, neutrophil count, NLR and CRP were higher in non-survivors, while lymphocyte count was lower in non-survivors.

**Table 4:** Comparison between survivors and non-survivors in COVID-19 patients with cancer

Clinical features	All patients (n = 103)	Survivor (n = 91)	Non-survivor (n = 12)	P-value
Age	66.0 (24.0–90.0)	66 (24.0–90.0)	66 (56.0–81.0)	0.487
Gender				0.061
Female	47 (45.6%)	45 (44.6%)	2 (16.7%)	
Male	56 (54.4%)	56 (55.4%)	10 (83.3%)	
Smoking	20 (19.4%)	16 (17.6%)	4 (33.3%)	0.242
Comorbidities				
Diabetes	14 (13.6%)	13 (14.3%)	1 (8.3%)	1.000
Hypertension	37 (35.9%)	36 (39.6%)	1 (8.3%)	0.052
Heart disease	11 (10.7%)	9 (9.9%)	2 (16.7%)	0.613
Symptoms				
Fever	73 (70.9%)	63 (69.2%)	10 (83.3%)	0.501
Cough	70 (68.0%)	63 (69.2%)	7 (58.3%)	0.183
Dyspnea	24 (23.3%)	16(17.6%)	8 (66.7%)	0.003
Vomiting	5 (4.9%)	4 (4.4%)	1 (8.3%)	0.34
Diarrhoea	7 (6.8%)	6 (6.7%)	1 (8.3%)	0.235
Laboratory findings				
WBC (× 109/L)	5.29 (1.90–25.10); n = 100	5.09 (1.90–25.10); n = 88	7.36 (3.50–18.80); n = 12	< 0.001
Neutrophil (× 109/L)	3.49 (0.67–22.40); n = 100	3.25 (0.67–22.40); n = 88	6.85 (1.50–17.36); n = 12	< 0.001
Lymphocyte (× 109/L)	1.06 (0.22–2.89); n = 100	1.15 (0.33–2.89); n = 88	0.64 (0.22–2.40); n = 12	0.012
NLR	2.93 (0.64–32.23); n = 100	2.64 (0.64–24.76); n = 88	11.7 (2.88–32.32); n = 12	< 0.001
CRP (mg/L)	2.52 (0.14–280.32); n = 90	1.79 (0.14–177.80); n = 81	80.00 (4.80–280.32); n = 9	< 0.001
ALT (U/L)	19.17 (4.50–131.20); n = 97	19.17 (4.50–131.20); n = 85	16.50 (6.00–68.30); n = 12	0.634
AST (U/L)	21.70 (10.00–147.20); n = 84	21.20 (10.00–147.20); n = 72	31.35 (12.00–92.6); n = 12	0.164

*WBC* white blood cell, *NLR* leukocyte to lymphocyte ratio, *CRP* C-reactive protein, *ALT* alanine transaminase, *AST* Aspartate transaminase

**Fig. 1:**
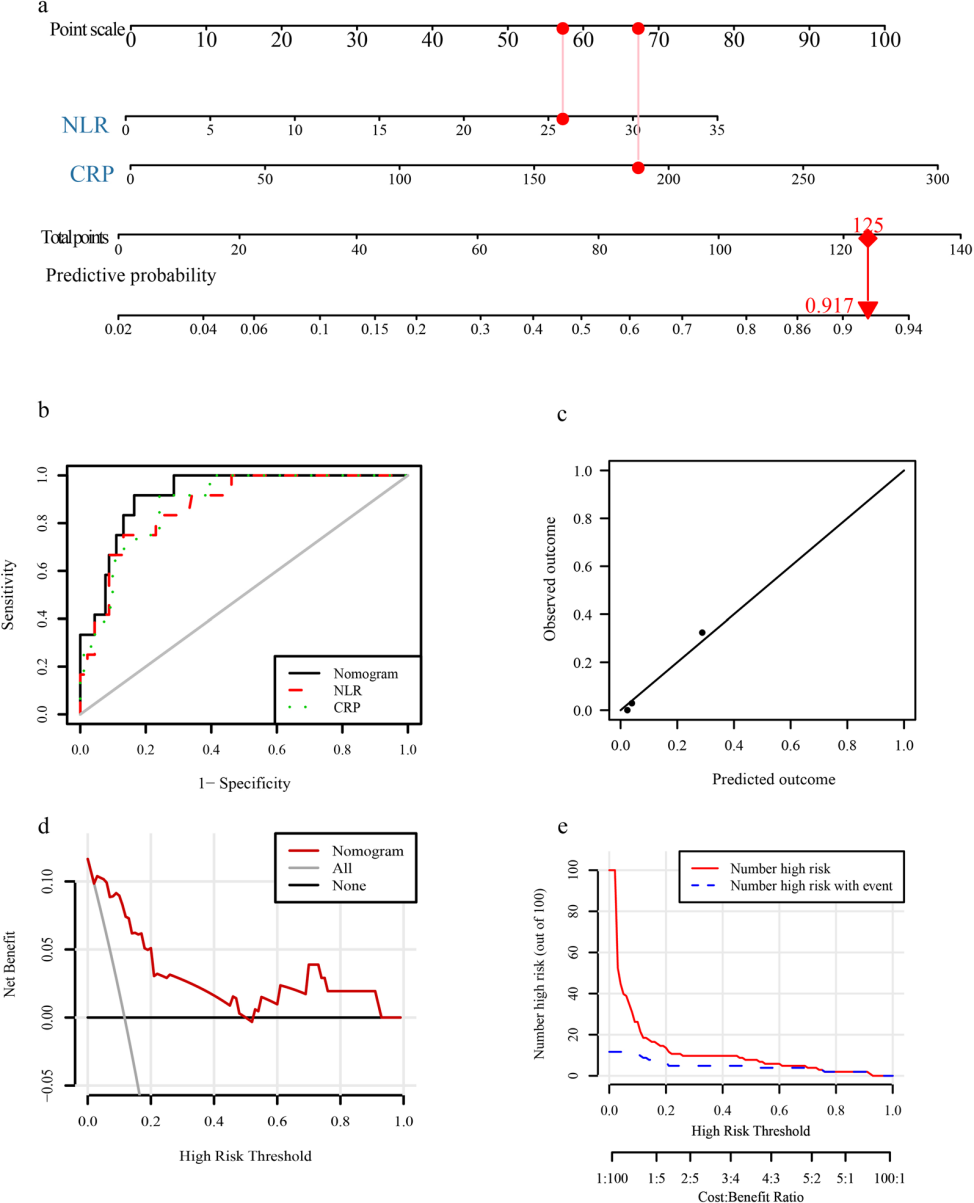
Development and validation of a nomogram. **a** A nomogram for predicting the probability of death. The observed value of each variable could get a matching point according to the point scale. Use the total points of all variables to evaluate the probability of death. **b** ROC curves for the nomogram, NLR and CRP. **c** Calibration plot, **d** decision curve and **e** clinical impact curve for the nomogram. ROC, receiver operating characteristic; NLR, neutrophil-to-lymphocyte ratio; CRP, C-reactive protein

Age, gender, smoking, number of comorbidities, number of symptoms, WBC, NLR, CRP, ALT, and AST were included in the generalized linear model. The results indicated that NLR and CRP were significant risk factors related to death outcome (Table 5). A nomogram to predict the probability of death was developed based on the two factors (Fig. 1a). The AUC of the nomogram was 0.918 (95% confidence interval [CI] 0.860–0.977), which was higher than that of NLR (0.872, 95%CI 0.784–0.961) and CRP (0.880, 95%CI 0.803–0.958) (Fig. 1b). The calibration plot showed that the predicted outcomes were in high agreement with observed outcomes (Fig. 1c). The decision curve and clinical impact curve showed that the nomogram had superior net benefit and influence on the death outcome of patients (Fig. 1d–e).

**Table 5:** P-value of each factor in the generalized linear model

Factors	Death outcome	Severe outcome
Age	0.990	0.514
Sex	0.130	0.340
Smoking	0.897	0.097
Number of comorbidities	0.130	0.279
Number of symptoms	0.565	0.552
WBC	0.318	0.469
NLR	0.033	0.040
CRP	0.040	0.082
ALT	0.588	0.451
AST	0.947	0.706

*WBC* white blood cell, *NLR* leukocyte to lymphocyte ratio, *CRP* C-reactive protein, *ALT* alanine transaminase, *AST* aspartate transaminase

For severe outcome, only NLR was significant factor in the generalized linear model (Table 5), hence we didn’t develop a nomogram for predicting the probability of severity.

## Discussion

The World Health Organization had declared COVID-19 to be a public health emergency of the world [11]. Patients with cancer are at higher risk of COVID-19, because they have low immunity and myelosuppressive effects caused by chemotherapy, surgery or radiotherapy for the cancer. Some studies reported that about 1–5.6% COVID-19 patients had cancer [9, 10, 13, 14]. In our study, we found that approximately 3.5% COVID-19 patients had cancer, which was consistent with that in previous reports.

A multicentre cohort study from China published earlier reported that COVID-19 patients with cancer had 24 different types of cancer, with breast cancer, colorectal cancer and lung cancer being the most common types [9]. Another small cohort study from China found that the most common cancer with COVID-19 was lung cancer, followed by breast cancer and rectal cancer [10]. In this study, we reported a total of 17 different types of cancer, of which breast cancer, lung cancer and bladder cancer were the three most common types of cancer.

The main symptoms of COVID-19 patients with cancer were fever, cough, dyspnea, vomiting, diarrhea, and so on, which were similar to those without cancer. When comparing cancer patients with severe COVID-19 with cancer patients with non-severe COVID-19, we found that the severe group had a higher median age and a higher proportion of smoker, diabetes, heart disease and dyspnea. Blood tests can real-time indicate the patient's condition. In severe group, the WBC count and neutrophil count were higher and lymphocyte count was lower, so the NLR is a significant factor to predict the severity of patients. We also found that the levels of pro-inflammatory cytokines and infection-related biomarkers were higher in severe COVID-19 patients with cancer, including procalcitonin, CRP and IL-6. In addition, the COVID-19 might injure the liver, heart, kidney and other vital organs. Hence, LDH, α-HBDH, D-dimer, NTproBNP, AST were higher in severe COVID-19 patients with cancer.

Wang and colleagues reported a much higher proportion of severe COVID-19 in cancer patients than in non-cancer patients [9]. But our study found that the severity rate in cancer patients was similar to that in non-cancer patients. The reason may be that the patients in our study were admitted to hospital after February 2020 and the patients reported in those studies were admitted to hospital mostly before February 2020. Patients included in this study received better treatment because of sufficient medical resources. So the severe rate of cancer patients in this study was lower than that in previous reports.

**Fig. 2:**
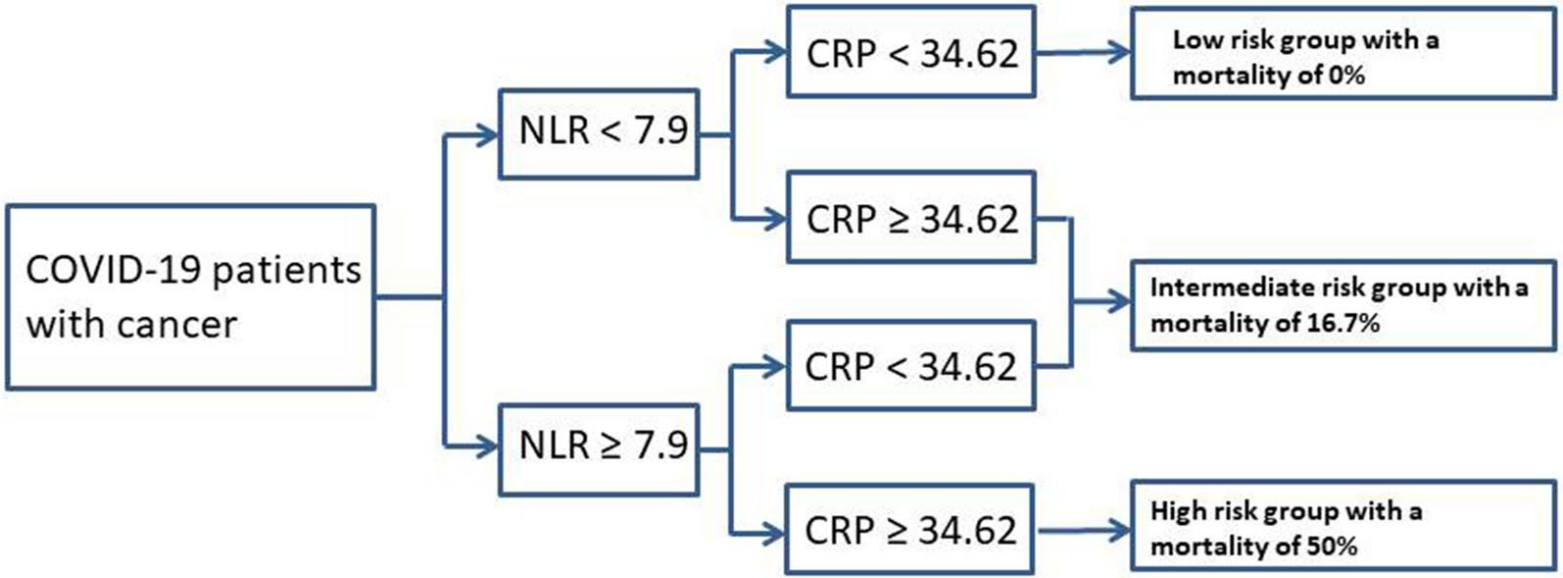
A chart of risk stratification by using neutrophil-to-lymphocyte ratio and C-reactive protein

The mortality in COVID-19 patients with cancer ranged from 15 to 28.6% in previous studies [9, 10, 15]. In this study, we reported a mortality of 11.7% in COVID-19 patients with cancer, which was higher than that in COVID-19 patients without cancer (4.4%). The global outbreak of COVID-19 brings huge challenges to the rational use of medical resources. Therefore, how to use effective markers to screen patients who need intensive care or have a high risk of death will help allocate medical resources effectively and reasonably, as well as reduce the mortality. Liang and his colleagues found that X-ray abnormality, age, hemoptysis, dyspnea, unconsciousness, number of comorbidities, cancer history, NLR, LDH and direct bilirubin at admission were significant predictors of critical illness, and developed a clinical tool with these 10 variables to predict the probability of developing critical illness for COVID-19 patients [16]. The tool had a good performance of AUC (0.88). Yan and his colleagues used LDH, lymphocytes and high-sensitivity CRP to develop a model to identify patients at high risk of death [17]. Liu reported that the severity of the patients with COVID-19 could be predicted by age and NLR [18]. In this study, we found that NLR and CRP were the most related risk factors with death outcome. A nomogram for predicting the probability of death based on these two factors was developed, with a high AUC of 0.918 and clinical benefit. An algorithm based on the nomogram (as presented in Figs. 1a and 2) could help physicians to screen high death risk COVID-19 patients with cancer on admission, and give effective preventive measures or intensive care. The cut-off value of NLR and CRP were 7.90 and 34.62, respectively. The patients with NLR < 7.90 and CRP < 34.62 had no risk of dying. The mortality risk of patients with NLR > 7.90 / CRP < 34.62 or NLR > 7.90 / CRP > 34.62 was 16.7%. However, more than 50% patients with NLR > 7.90 and CRP > 34.62 died (Fig. 2). The risk stratification would help us to take care of the high-risk patients and reduce the mortality.

There were several limitations in this study. First, the clinical records were incomplete. Especially no dynamic changes of laboratory results were recorded, which might help us to better identify severity or death outcome related risk factors. Second, the sample of COVID-19 patient with cancer was limited. No external validation of the nomogram was performed.

## Conclusion

This study presented the details of clinical features and laboratory results in 103 COVID-19 patients with cancer. The main cancer categories were breast cancer, lung cancer, bladder cancer. The mortality was 11.7%. In addition, this study found that NLR and CRP were death risk factors. An algorithm based on the two factors could help to screen high risk patients and give adequate treatment and protective measures in advance.

## Supplementary Information

**Additional file 1:** **Figure S1.** A flow chart of patients selection.

## Data Availability

The datasets used and/or analysed during the current study are available from the corresponding author on reasonable request.

## Abbreviations

COVID-19: Coronavirus disease 2019
AUC: Area under the curve
ROC: Receiver operating characteristic
IQR: Interquartile range
WBC: White blood cell
NLR: Neutrophil-to-lymphocyte ratio
CRP: C-reactive protein
IL-6: Interleukin-6
LDH: Lactate dehydrogenase
αHBDH: Alpha-hydroxybutyric dehydrogenase
NTproBNP: N-terminal pro brain natriuretic peptide
AST: Aspartate transaminase
ALT: Alanine transaminase
