# Supplementary figures and images for: Clinical features and death risk factors in COVID-19 patients with cancer: a retrospective study
Source: BMC Infect Dis. 2021 Aug 5;21:760. doi: 10.1186/s12879-021-06495-9 (PMC8340077; doi:10.1186/s12879-021-06495-9)

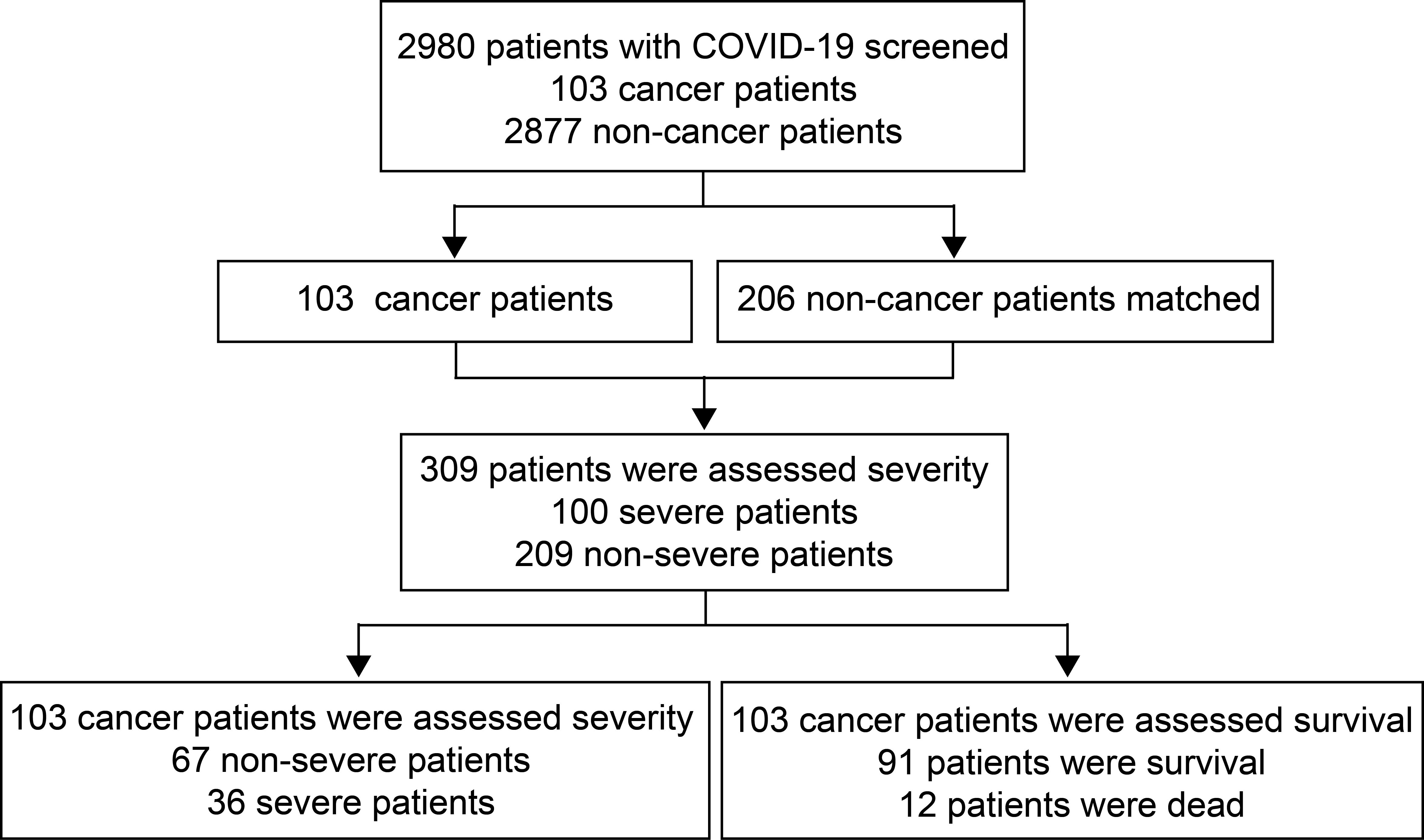

Supplement: Supplementary file 1 — Additional file 1: Figure S1. A flow chart of patients selection. [file 12879_2021_6495_MOESM1_ESM.jpg]
